# Different behavior of myeloperoxidase in two rodent amoebic liver abscess models

**DOI:** 10.1371/journal.pone.0182480

**Published:** 2017-08-10

**Authors:** Andrea Cruz-Baquero, Luz María Cárdenas Jaramillo, Manuel Gutiérrez-Meza, Rosa Adriana Jarillo-Luna, Rafael Campos-Rodríguez, Víctor Rivera-Aguilar, Angel Miliar-García, Judith Pacheco-Yepez

**Affiliations:** 1 Sección de Estudios de Posgrado e Investigación, Escuela Superior de Medicina, Instituto Politécnico Nacional, Plan de San Luís y Díaz Mirón, CP, Ciudad de México, México; 2 Coordinación de Ciencias Morfológicas, Escuela Superior de Medicina, Instituto Politécnico Nacional, Plan de San Luís y Díaz Mirón, CP, Ciudad de México, México; 3 Departamento de Microbiología, UBIPRO, FES-Iztacala, UNAM, CP, Tlanepantla, Estado de México, México; Universita degli Studi di Parma, ITALY

## Abstract

The protozoan *Entamoeba histolytica* is the etiological agent of amoebiasis, which can spread to the liver and form amoebic liver abscesses. Histological studies conducted with resistant and susceptible models of amoebic liver abscesses (ALAs) have established that neutrophils are the first cells to contact invasive amoebae at the lesion site. Myeloperoxidase is the most abundant enzyme secreted by neutrophils. It uses hydrogen peroxide secreted by the same cells to oxidize chloride ions and produce hypochlorous acid, which is the most efficient microbicidal system of neutrophils. In a previous report, our group demonstrated that myeloperoxidase presents amoebicidal activity *in vitro*. The aim of the current contribution was to analyze *in vivo* the role of myeloperoxidase in a susceptible (hamsters) and resistant (Balb/c mice) animal models of ALAs. In liver samples of hamsters and mice inoculated intraportally with *Entamoeba histolytica* trophozoites, the number of neutrophils in ALAs was determined by enzymatic activity. The presence of myeloperoxidase was observed by staining, and its expression and activity were quantified *in situ*. A significant difference existed between the two animal models in the number of neutrophils and the expression and activity of myeloperoxidase, which may explain the distinct evolution of amoebic liver abscesses. Hamsters and mice were treated with an MPO inhibitor (4-aminobenzoic acid hydrazide). Hamsters treated with ABAH showed no significant differences in the percentage of lesions or in the percentage of amoebae damaged compared with the untreated hamsters. ABAH treated mice versus untreated mice showed larger abscesses and a decreased percentage of damaged amoebae in these lesion at all stages of evolution. Further studies are needed to elucidate the host and amoebic mechanisms involved in the adequate or inadequate activation and modulation of myeloperoxidase.

## Introduction

The protozoan *Entamoeba histolytica* (*E*. *histolytica*) is the etiological agent of amoebiasis, which mainly affects the colon. Under some circumstances, it can spread to the liver and gives rise to amoebic liver abscess (ALAs)[1] [2]. It is known that there is a gender-based susceptibility to the development of invasive amoebiasis, being more frequent for adult males. This may be due to a greater male-related susceptibility to asymptomatic infectious [3]. Gender differences have also been observed in animal models, whereas female C57BL/6 mice were resistant to ALA development, male mice presented a prolonged ALA recovery time [4]. However, the reasons for these differences are still unknown.

*In vitro* and *in vivo* studies have demonstrated that at liver lesion areas, the first line of immune defense against *E*. *histolytica* trophozoites is represented by neutrophils, which produce and release reactive oxygen species (ROS) and antimicrobial peptides [5]. Regarding in vivo studies, resistant (mice) and susceptible (hamster or gerbil) animal models to ALA have been widely used [6], preferentially with males to avoid the hormonal effect. A different activity and amoebicidal effect of neutrophils has been found in resistant and susceptible animal models, thus generating distinct pathogenesis of ALA development.

In hamsters (*Mesocricetus auratus*), inoculation with *E*. *histolytica* induces a rapid inflammatory infiltrate, mainly compose by neutrophils. These cells sorround trophozoites and isolate them from the hepatic parenchyma. Afterwards, there is a significant lysis of neutrophils, responsible in part for the parenchymal damage during ALA evolution. This pathogenesis leads to more than 90% mortality in hamsters [7].

In Balb/c mice, neutrophils arrive at the liver at 3 hours post-intraportal inoculation with parasites [8] and the damage is produced to the liver parenchyma at 12 hours. Ischemia areas present zones surrounded by an acute inflammatory infiltrate composed of polymorphonuclear cells (PMNs), mainly neutrophils. The trophozoites are totally eliminated at the fourth day after-inoculation; the regenerative process appears posteriorly in the liver.

In a study on SCID mice with ALA, hepatic abscesses reached a greater size when the animals were depleted of neutrophils, indicating a protective role exerted by these cells in the defense against *E*. *histolytica* [9]. More recently, a study reported that in a mouse model of ALA at day one post-infection, the neutrophils in the ALA are dispensable for the control of the abscess, do not have a beneficial role in the resolution of ALA and are not critical to the tissue damage [10]. Hence, the role of these cells in the development of ALA is still not well defined.

In contrast, neutrophils directly contribute to tissue damage in susceptible species (gerbils and hamsters), [11] while they effectively control and resolve the infection in resistant species (mice, rats and guinea pigs) [8]. The distinct outcome may depend on a different degree of activation and efficiency of the microbicidal mechanisms of neutrophils.

One important mechanism is the release of myeloperoxidase (MPO), an enzyme present in the azurophilic granules of neutrophils and in primary lysozymes of monocytes [12–21]. When neutrophils are activated, MPO is released inside the phagosome during the phagocytosis of microorganisms [22, 23]. The substrate of MPO is hydrogen peroxide (H_2_O_2_), which results from the reaction between the molecular oxygen and NADPH oxidase complex [24]. MPO utilizes H_2_O_2_ to catalyze the oxidation of two ions of chloride (Cl^-^) and produce hypochlorous acid (HOCl) [25]. By modifying cellular lipids and proteins, this oxidizing agent causes tissue damage at a local level and thus amplifies the inflammatory cascade [26].

Diverse studies have demonstrated MPO activity in chronic inflammatory diseases such as cystic fibrosis and other autoimmune diseases [27–29]. Additionally, abundant evidence exists regarding the participation of MPO in the host defense against invasion by bacteria, fungi and viruses [30–36]. It follows that individuals with MPO deficiency have a greater incidence of infection [37] [38]. Specifically, the binding of neutrophil MPO to the microbial surface is the first step in microbicidal activity, according to observations from experiments with *Actinobacillus actinomycetemcomitans* [39]. Moreover, purified MPO is toxic for *Trichinella spiralis* [40] and *Schistosoma mansoni* [41]. Several studies have concluded that MPO not only eliminates intracellular pathogens but also acts against extracellular organisms [38, 40, 41].

The aim of the current contribution was to analyze the role of the MPO enzyme at different stages of ALA evolution in a susceptible (hamsters) and resistant (Balb/c mice) animal model. The results showed that during the pathogenesis of ALA, there is a distinct role of MPO in each of these models. Further research is necessary to elucidate the modulation of MPO enzyme in the host-parasite relationship in an in vivo model.

## Materials and methods

### Amoebic cultures

*Entamoeba histolytica* trophozoites of the HM-1:IMSS strain were cultured axenically at 37°C in Diamond trypticase yeast iron extract (TYI-S-33) culture medium supplemented with 20% bovine serum (GIBCO BRL, USA) [42]. The trophozoites were harvested at the end of the logarithmic growth phase (48 h) by chilling to 4°C. They were concentrated by centrifugation at 300 g for 5 min and used immediately.

### Animals

Male Balb/c mice (weighing approximately 30 g) were employed for the resistant model and male hamsters (*Mesocricetus auratus*; weighing approximately 100 g) for the susceptible model. In each case, animals were randomly distributed into 5 groups (n = 10): the uninfected control (CT) and the four experimental groups inoculated with *E*. *histolytica* and sacrificed at either 3, 6, 12 or 24 h post-infection. The current protocol was developed based on the ARRIVE guidelines for reporting animal research [43], and was approved by the Ethics in Research Committee of the Escuela Superior de Medicina (IPN). Animals were handled in accordance with Mexican federal regulations for animal experimentation and care (NOM-062-ZOO-1999, Ministry of Agriculture, Mexico City, Mexico).

### Inducing ALA in animals and collecting tissue

Male Balb/c mice and male hamsters were anesthetized intraperitoneally with 1.3 mg of sodium pentobarbital (0.5 mg/100 g) (Atitalaquia, Hgo, Mexico). After a laparotomy, they were inoculated intraportally with 1 X 10^6^ trophozoites in 100 μl of culture medium. The animals were sacrificed at 3, 6, 12 and 24 h post-inoculation [7]. Representative fragments of the liver lesions were processed for paraffin embedding, from wich 7 μm cuts were made [44], placed on cover slips and stained with hematoxylin and eosin (H & E). The samples were analyzed by microscopy, and the most representative samples were selected to perform histochemical and immunohistochemical stains. A representative image of the samples selected is shown in S1 Fig. Samples were frozen to -80°C in phosphate-buffered saline for MPO activity assays and others in Trizol for MPO expression assays.

### Quantitative evaluation of neutrophils in ALA

To assess neutrophil accumulation in the ALAs of tissue samples, the histochemical reaction was induced by AS-D chloroacetate esterase with an Esterase Kit, following the manufacturer’s instructions (Sigma, St. Louis, MO, USA). Using the Image-Pro Plus 5.1 software and an E600 microscope (Nikon, Eclipse E-600, Japan) with 40x magnification, the total number of cells and the number of cells testing positive for AS-D chloroacetate esterase (neutrophils) [45] were counted in the inflammatory infiltrates (8 per slide/3 slides per animal, n = 5). Then, the percentage of neutrophils versus total cells was calculated for each animal.

### MPO immunohistochemistry

After samples of liver lesions were fixed with paraformaldehyde and embedded in paraffin, 7-μm cuts were made. The samples were deparaffinized and hydrated with PBS. Antigenic recovery was carried out in citrate buffer (10mM, pH 6.0, 0.05% Tween-20) at 90°C for 10 minutes. Subsequently, samples were chilled and washed with citrate buffer for 20 minutes and then with PBS. Endogenous peroxidase was inhibited with 3% H_2_O_2_ in PBS for 30 minutes and then blocked with FBS at 3%. The slides were incubated with D Avidin solution (Vector Laboratories, SP-2001, Burlingame, CA, USA), washed with PBS, incubated with biotin solution for 15 min, and then washed again with PBS. Samples were labeled with the primary antibody, 1μg/ml rabbit polyclonal anti-MPO (ab9535, Abcam, Cambridge, UK), and incubated overnight at 4°C in a humid chamber. Afterwards, they were incubated with the secondary antibody, Biotin-Goat anti-rabbit IgG H+L DS Grade (Invitrogen, ZYMED, CA, USA), at 3 μg/ml for 1 h, followed by incubation with streptavidin-biotin peroxidase (ZYMED, CA, USA) at 2.5 μg/ml for 1 h. Finally, diaminobenzidine at a dilution of 1:9 (Kit DAB Thermo, Waltham, MA, USA) was added to the samples, which were subsequently counterstained with hematoxylin (1:9) and washed for 1–2 min with tap water and then with distilled water. Finally, the samples were dehydrated in alcohol and xylene and analyzed by microscopy (NIKON Eclipse E600, Japan) [7, 8, 44]. Control staining was performed following the same protocol, except that the primary antibody was substituted with an irrelevant antibody, anti-human S-100 protein, rabbit polyclonal IgG (Santa Cruz SC7849-1 CA, USA).

#### Immunofluorescense of *E*. *histolytica* trophozoites and MPO

Samples of hamster ALA (6 h) embedded in paraffin were labeled with primary polyclonal antibody rabbit anti-MPO (1μg/ml; ab9535, Abcam, Cambridge, UK) and incubated overnight at 4°C. Afterwards, samples were incubated with the secondary antibody goat anti- rabbitt IgG-FITC (USBiological Catalog No 1193-62G). Then, they were incubated with rabbit IgG anti-*E*. *histolytica* (manufactured in our laboratory) for 2 h at RT. Subsequently, they were incubated with a secondary antibody goat anti-rabbitt IgG-Rhodamine (USBiological Catalog No 1103-19P7) for 1 h. Finally, the slides were incubated with Sudan black 0.05% in an ethanol 70% solution for 30 min and observed with fluorescence microscopy (Nikon, Eclipse CI H550S, Japan).

### Quantitative analysis of MPO-positive cells in ALAs

For the immunohistochemical samples that were stained for MPO, a count was made of the positive cells in three fields of ALA lesions for each hamster and five fields for each mouse (in each case with a 0.05 mm^2^ field) at 40X (NIKON Eclipse E600, Japan). The number of cells counted was evaluated with the Image-Pro®Plus version 5.1 program. The data were processed in Excel and analyzed with SigmaPlot 11.

### RNA extraction and real-time qPCR

Expression of *mpo* genes in both the resistant and susceptible models was determined in total RNA from liver lesions. Briefly, total RNA was extracted by the Trizol method (TRIzol, Ambion, Van Way, Carlsbad, CA, USA). At the end of the process, the supernatant was removed and allowed to dry at room temperature, then resuspended with RNAase-free water and homogenized. Quantification was carried out using Nanodrop Lite (Thermo scientific, Waltham, MA, USA). RNA integrity was evaluated by performing electrophoresis of 5 μg on 1% agarose gel containing ethidium bromide, followed by analysis in a UV transilluminator (Epi Chemi II Darkroom UVP, Laboratory Products, Upland, CA, USA). RNA was stored at -80 ^o^C and on the following day, 1 μg of total RNA was reverse-transcribed in a 20 μl reaction volume with a Transcriptor First Strand cDNA Synthesis Kit (Roche Indianapolis, IN, USA). The reactions were conducted in a thermocycler (Mastercycler gradient, Eppendorf, Hamburg, Germany), and the protocol was based on the manufacturer’s recommendations. Specific oligonucleotide primers were generated with the online assay design software (probeFinder: http://www.universalprobelybrary.com).

The primer sequences for the mouse *mpo gene* were as follow: *mpo*F 5´ TCCCGGTATGTGATGATCTG 3´ and *mpo*R 5´ TCGATGGAATGGGGAGAA 3´. Expression of the *mpo* gene in hamsters (Gene ID: 101830171) was determined by utilizing a prediction of the *mpo* gene sequences found in the NCBI database. The MPO gene in mice (Gen ID: 17523), the mouse *gapdh* gene (Gen ID: 14433) and the hamster *gapdh* gene (Gen ID: 106022412) were taken from the same database. Specific oligonucleotide primers were generated using the aforementioned method, and primer sequences employed for hamsters were *mpo*F 5´ GCTGTGCACTGAACACACCT 3´ and *mpo*R 5´ TTTAGGAAGCTACGGGATGG´. The 20 μl reaction mixture contained 1 x LightCyclerTaqMan Master reaction mixture (Roche Diagnostics, Indianapolis, IN, USA), 200 nM of each primer, 100nM of Universal -ProbeLibrary probe, 0.5 U LightCycler Uracil-DNA Glycosylase and 2 μl of standard DNA in the appropriate dilution. The amplification was performed on 0.2 ml tube strips (Roche Diagnostics, Indianapolis, IN, USA). The RT-qPCR assays were carried out in a LightCycler®Nano SW 1.1 Instrument (Roche Diagnostics Indianapolis, IN, USA), and data were analyzed with LightCycler Software. Finally, mRNA levels were calculated by employing the comparative parameter quantification cycle (Cq) method with reference genes (the efficacy of the PCR was 100%) [46]. The primer sequences for *gapdh* were *gapdh*F 5´TTTGATGTTAGTGGGGTCTCG 3´ and *gapdh*R 5´ AGCTTGTCATCAACGGGAAG 3´ for mice and *gapdh*F 5´ GGCAACAACTTCCACTTTGC 3´ and *gapdh*R 5´ CGTATTGGACGCCTGGTTAC 3´ for hamsters.

#### MPO chlorination activity

A 100-mg portion from representative ALAs was homogenized in 500 μl of cold PBS and sonicated on ice. Subsequently, the samples were centrifuged at 10,000 g and 4°C for 15 min. The supernatant was removed, placed on ice, and then stored at -80°C to await further use.

For Cayman’s myeloperoxidase chlorination assay (Ann Arbor, MI, USA), we followed the manufacturer’s recommendations. Briefly, 50 μl of the sample was added to each well, along with the MPO inhibitor diluted 1:2. Samples were prepared in duplicate with 2- [6- (4-aminofenoxi) -3-oxo-3H-xanten-9-il] benzoic acid (APF), which was selectively split with hypochlorite (-OCl) to produce fluorescein, a highly fluorescent compound. The results were read with a 96-well fluorescence reader (Biotek synergy, Winooski, VT, USA). Fluorescence was analyzed with an excitation wave length of 480 to 495 nm and an emission wave length of 515 to 525 nm to determine relative fluorescence units (RFU).

For calculating enzymatic activity, the following equation was used:

$$\frac{RFU}{min}=\frac{RFU\;(time\;2)–RFU\;(time\;1)}{Time\;2\;(min)-Time\;1\;(min)}$$

where times 1 and 2 are expressed in minutes.

To obtain MPO activity, the following equation was employed [17, 47]:

$$\mathrm{Myeloperoxidase}\;\mathrm{activity}\;\left(\mathrm{pmol}/\min/\mathrm{ml}\right)=\frac{sample\;slope\;(\frac{RFU}{min})-Inhibitor\;slope\;(\frac{RFU}{min})}{Fluorescein\;standard\;curve\;slope}\;x\;Sample\;dilution$$

#### Treatment of hamsters and Balb/c mice with 4-aminobenzoic acid hydrazide, followed by *E*. *histolytica* inoculation

Stock solutions of 10 mg of 4-aminobenzoic hydrazide (ABAH) (Aldrich Chemistry, St Louis, MO, USA) were prepared in 50 μl DMSO plus 950 μl Milli-Q water. Hamsters (9 weeks of age, weighing approximately 100 g) and male BALB/c mice (7–10 weeks of age, weighing approximately 30 g) were treated with ABAH at 40 mg/Kg every 12 h [48]. After five days of the ABAH treatment, animals were anesthetized with sodium pentobarbital, as previously mentioned, and a laparotomy was practiced under aseptic conditions. Control hamsters and mice were treated with PBS or DMSO. Subsequently, hamsters, mice and control groups were inoculated with 1x10^6^ *E*. *histolytica* trophozoites by the intraportal route, and then the abdominal layers were sutured with surgical staples. Hamsters and Balb/c mice were sacrificed at 3, 6 and 12 h post-inoculation. Whole liver abscesses were dissected and weighed to evaluate the percentage of the organ occupied by amoebic lesions. This percentage was calculated as the weight of the abscesses divided by total liver weight (recorded before abscess removal). With these ALA hamsters and mouse samples labeled for MPO, damaged and undamaged amoebae were quantified by examining morphological changes/alterations in the lesion areas (n = 6). Quantification was made using Image-Pro Plus 5.1 software in an E600 microscope (Nikon, Eclipse E-600 Japan) with 40x magnification. The total number of damage and undamaged amoebae in the ALA were counted in the lesion areas (n = 6).

### Statistical analysis

Data represent the consensus of three independent experiments and are expressed as the mean ± SD. The comparison between 2 groups was analyzed using Student’s unpaired two-tailed *t-*test. For a comparison between more than two groups, one-way ANOVA was employed. If a significant (*p*<0.05) main effect or association was identified, the respective group means were compared using the Bonferroni test. All analyses were performed with the Graph Pad Prism software (version 6).

## Results

### Neutrophils positive for AS-D esterase are in the ALAs of hamster and mouse models

**Fig 1:**
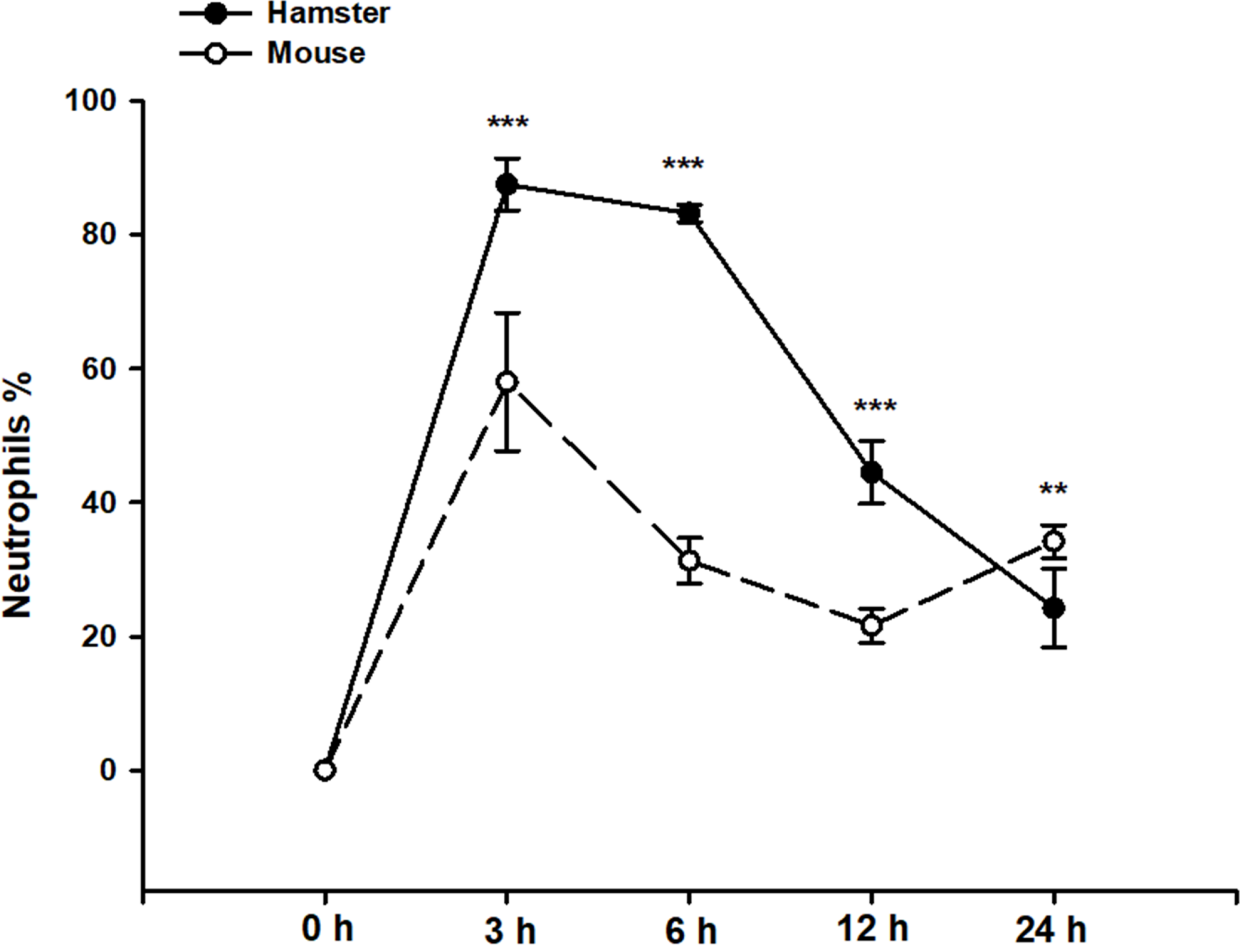
Percentage of neutrophils stained by AS-D esterase in ALAs from hamsters and Balb/c mice. Liver sections were stained with AS-D chloroacetate esterase. Neutrophils were identified by their well-known morphology and a positive stain. The total number of cells were counted in the inflammatory focus (8 infiltrates per slide/3 slides per animal), as were the number of cells positive to AS-D chloroacetate esterase (neutrophils) by using Image-Pro Plus 5.1 with 40x magnification. The percentage of neutrophils was calculated. During the evolution of hamster ALA, the percentage of neutrophils diminished progressively. In mice, the percentage of neutrophils was less compare with the hamsters but the changes showed similar behaviour, except at 24 hours. At the latter times post infection, the percentage of neutrophils was higher in mice than hamsters. Data represent the mean ± SD of the three independents experiments, (n = 5). *P*-values were determined by the Student’s *t*-test (*** p<0.001; ** p<0.01).

To determine neutrophil infiltration, sections of ALAs from hamsters and Balb/c mice sacrificed at 3, 6, 12 and 24 h post-inoculation and a specific marker for neutrophils (AS-D chloroacetate esterase) were used. The uninfected groups did not present inflammatory infiltrates. In hamster ALAs, the number of AS-D esterase-positive cells reached a peak at 3 h post-inoculation (85%) (Fig 1), then decrease with evolution of ALA. The lowest number of positive cells was found at 24 h (24.2%). The number of neutrophils in mouse ALAs was the highest at 3 h (58%) of evolution. There was a decrease in the number of neutrophils at 6 h (31%) and 12 h (21.6%) of ALA evolution, followed by a slight increment at 24 h (34.25%) (Fig 1). The increase in the neutrophils in the infiltrates and the posterior reduction in the percentage was quite similar in both animal models, except that the reduction of neutrophils was more drastic in hamsters than in mice, as determined by Student’s *t*-test (*** p<0.001; ** p<0.01).

**Fig 2:**
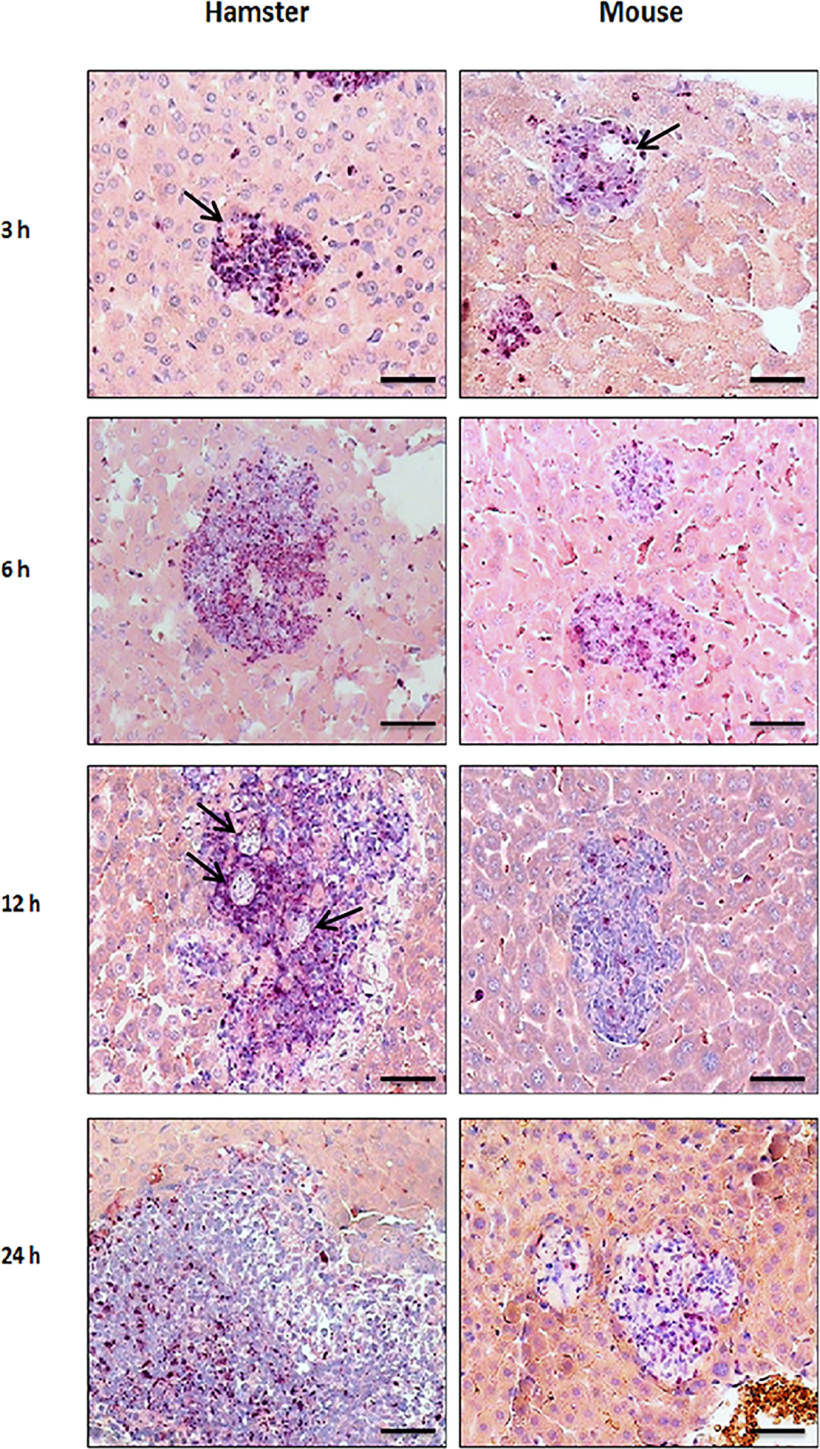
Neutrophils positive to esterase in hamster and mouse ALAs. Liver tissue was processed by histochemistry. Neutrophils were positive to AS-D chloroacetate esterase in ALA from hamsters and mice at 3, 6, 12 and 24 h post inoculation. Amoebae are present inside the inflammatory infiltrate (arrows). Barr = 50μm.

Our results showed that neutrophils were more abundant in hamsters than in mice in ALA development (Fig 2). A higher magnification of AS-D esterase staining in hamster and Balb/c mice at 6 h revealed a typical morphology of neutrophils with a granular label for AS-D esterase (S2 Fig). Our results showed that positive neutrophils are more abundant in the hamsters, as demonstrated previously; however, the labeling in the mice was quite similar. The percentage of neutrophils versus the total number of cells was higher in hamsters than mice, except at 24 h post-inoculation when the ratio in the percentage of cells is reverse (Fig 1).

### Neutrophils positive for MPO in hamster ALAs

**Fig 3:**
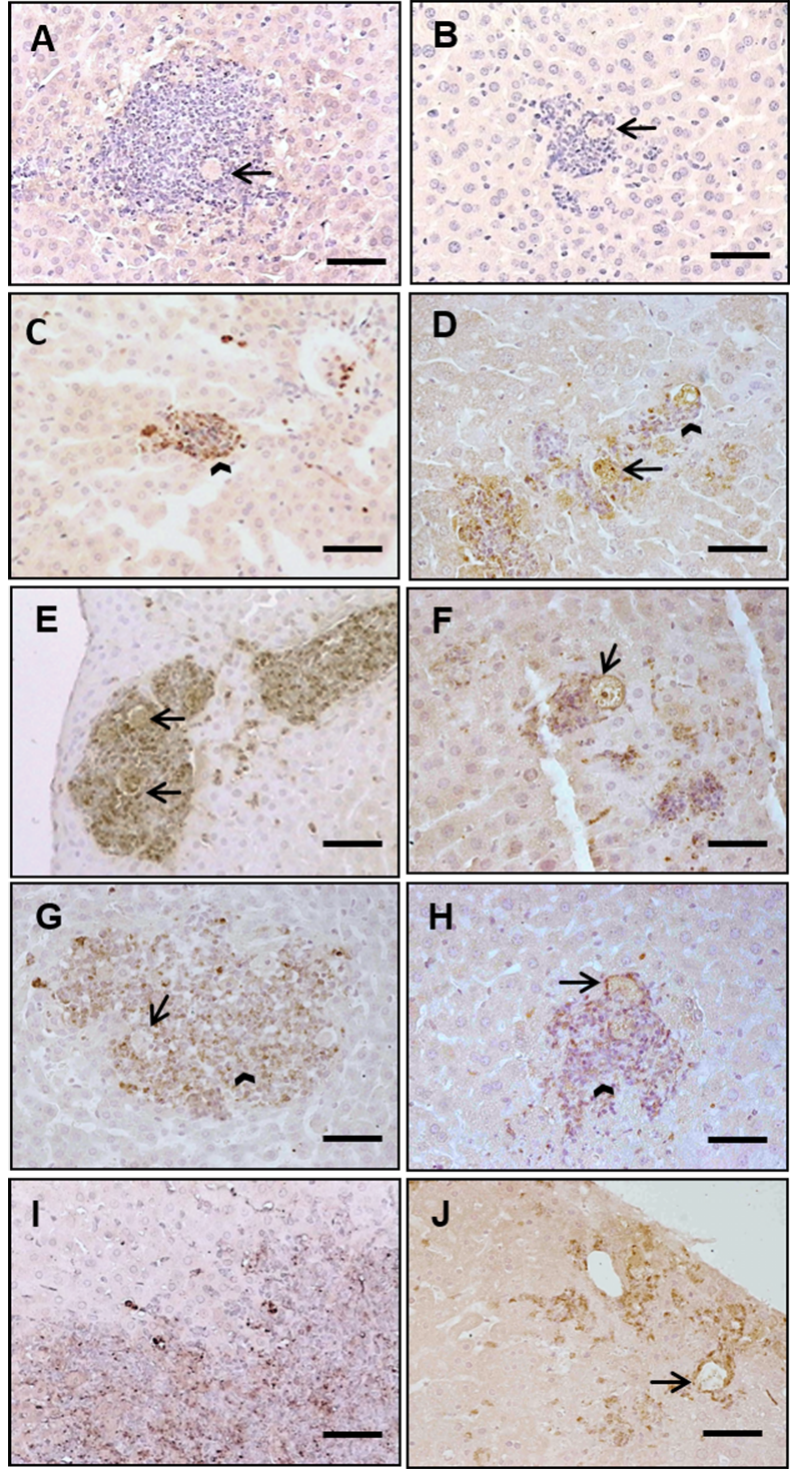
Neutrophils positive to MPO in hamster and mice ALAs. Liver tissue was processed by immunohistochemistry to detect the presence of MPO in ALA of hamsters (A, C, E, G, I) and mice (B, D, F, H, J) at 3, 6, 12 and 24 h post-inoculation. Negative controls was performed with an irrelevant, antibody no label was observed (A, B). (C) Hamster liver lesions, neutrophils were positive in small inflammatory foci at 3 h post-inoculation (arrowhead). (E) At 6 h post-inoculation, amoebae (arrows) were positive to MPO. (G) At 12 h, appears extensive inflammatory reaction composed by damaged neutrophils positive to MPO. (I) At 24 h, inflammatory cells lysed on the border of a necrotic area were also stained to MPO. (D) Mouse liver lesions; inflammatory cells showed MPO label at 3 h post-inoculation (arrowhead), amoeba is seen (arrow). (F) At 6 h post-inoculation, neutrophils appear surround the damaged amoeba (arrow). (H) At 12 h appear damaged amoeba (arrow); the inflammatory infiltrate was constituted by neutrophils positive to MPO (arrowhead). (J) At 24 h staining for MPO was evident, the amoeba present signs of damage (arrow). Barr = 50μm.

Hamster liver abscess were analyzed at 3, 6, 12 and 24 h post-inoculation. Using immunohistochemistry, we evaluated the presence of MPO in the lesions. Negative controls were incubated with an irrelevant antibody; this control did not present positive staining for MPO (Fig 3A). A small inflammatory reaction showed that neutrophils were positive for MPO at 3 h post-inoculation (Fig 3C). At 6 h post-inoculation, large inflammatory foci with numerous neutrophils were positive for MPO staining (Fig 3E). The hepatocytes bordering the inflammatory foci showed signs of damage. At 12 h, extensive inflammatory infiltrates were observed; some neutrophils appear lysed but were positive to the MPO (Fig 3G). The lysis of the inflammatory cells with the consequent release of MPO could be participating in the tissue damage. At 24 h, the liver damage was more evident, the majority of the inflammatory cells appear lysed, and the necrotic area was stained for MPO (Fig 3I). Additionally, a representative ALA lesion (6 h) was examined with the aim of observing the localization of *E*. *histolytica* trophozoites and MPO using an immunofluorescence assay. MPO localization was positive in the inflammatory reaction and surrounded the amoebae. The hepatocytes were negative to MPO (S3 Fig).

### Neutrophils positive for MPO surrounded damaged amoebae in mice with ALAs

Mouse liver abscess were analyzed at 3, 6, 12 and 24 h post-inoculation. Liver tissue was processed by immunohistochemistry with the purpose of evaluating the presence of MPO in the ALA. An irrelevant antibody was used for control samples (Fig 3B), and no labeling was found for hepatocytes, *E*. *histolytica* or the inflammatory focus. Regarding the experimental groups, inflammatory areas mainly comprised neutrophils at 3 h post-inoculation, and some of these cells were positive for MPO. Moreover, positive staining for neutrophils was observed inside in the trophozoites (Fig 3D). At 6 h, lysed neutrophils appear surrounding the damaged trophozoites (Fig 3F). At 12 h, the inflammatory area was comprised MPO-positive polymorphonuclear cells that appear in close contact with the amoebae, displaying a slight staining for MPO (Fig 3H). At 24 h, the damaged area near to the Glisson capsule showed staining for MPO, which was mainly present in the damaged liver tissue (Fig 3J).

### Cells positive for MPO in ALA development in hamsters and mice

**Fig 4:**
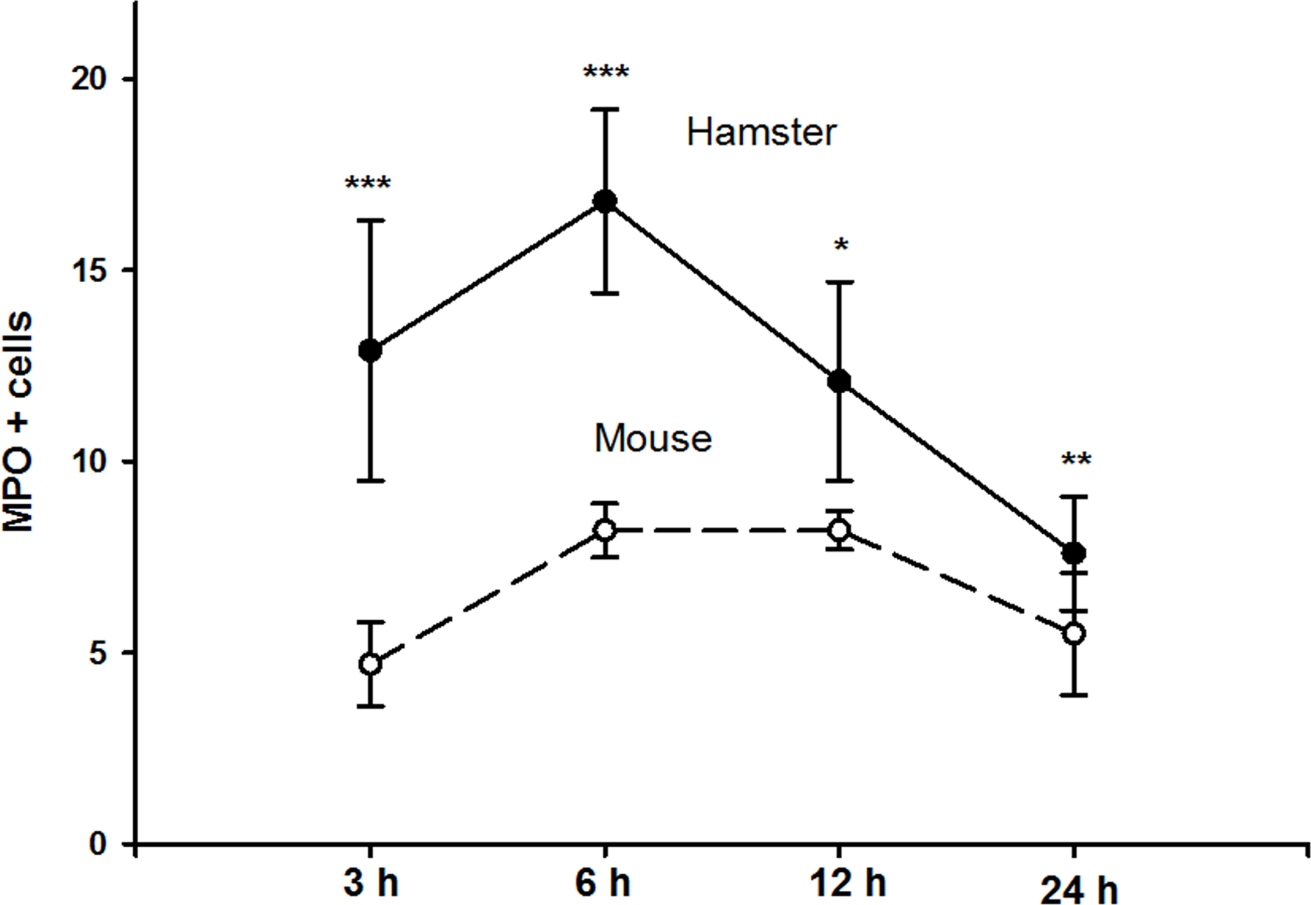
Different kinetics of cells positive to MPO in hamster and mouse ALAs model. Hamsters showed an increase in neutrophils positive to MPO at 3 and 6 h, with a reduction in the number of cell at 12 and 24 h. In mice the MPO was positive at 6 and 12 h and decrease importantly at 24 h. Data represent the mean ± SD of three independent experiments (n = 5). *P*-values were determined by the Student’s *t*-test (*** p<0.001; ** p<0.01; * p<0.05).

Hepatic lesions in hamsters presented an increase in the number of cells positive for MPO at 6 h post-inoculation (ANOVA, 3(2, 16) = 9.5; p<0.001), followed by a decrease at 12 and 24 h (Fig 4) (p<0.005). In the mouse model, hepatic lesions had an increase in the number of MPO-positive cells (one-way ANOVA, 3(2, 16) = 13.5; (p<0.001) at 6 and 12 h post-inoculation (Fig 4) (p<0.001). Significant differences were observed in the number of cells that were positive for MPO in mice and hamsters at 3, 6 and 12 h (p<0.001, p<0.001 and p = 0.041, respectively; Student’s *t*-test), but not at 24 h (p = 0.292).

The number of MPO-positive cells at different time periods was different in the two rodent models. There were more positive cells in hamsters compared with mice throughout the evolution of ALA. It is important to mention that in mice, the number of positive cells was maintained at 6 and 12 h, and these cells can be activated and produces MPO, which could be contributing to *E*. *histolytica* trophozoites damage.

### Expression of the *mpo* gene decreased in hamster ALAs

**Fig 5:**
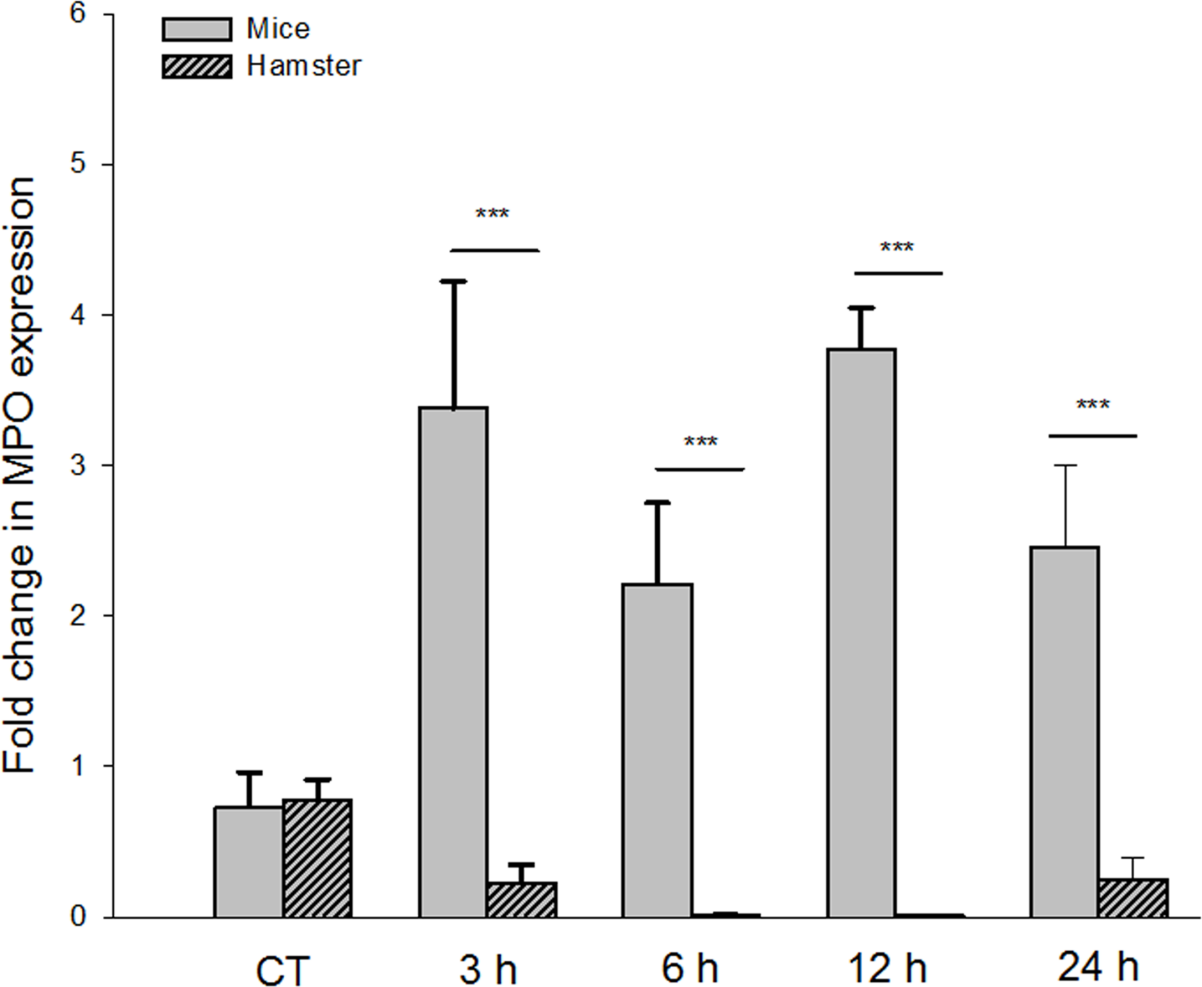
Differential expression of the *mpo* gene in hamster and mice ALAs. Mice tissue samples show a significant high expression of the *mpo* gene at all post-inoculation times compared with the control group. The difference between these two ALAs models were higher at 3 and 12 h (p<0.001). Hamsters ALA did not show expression of the *mpo* gene compared with the control group, the lowest expression was found at 6 and 12 h (p<0.001). There is a statistically significant difference between the species and post-inoculation time (p<0.001). Data represent the mean ± SD of three independent experiments, (n = 5). *P*-values were determined by the one-way ANOVA (*** p<0.001).

Compared to the control group, *mpo* expression was ~0.245-fold lower at 3 and 24 h post-inoculation (p< 0.001) and an additional ~0.015-fold lower at 6 and 12 h. As can be appreciated, there was a significant reduction in *mpo* expression after *E*. *histolytica* was inoculated in the susceptible model of ALA, with the lowest values of this parameter found at 6 and 12 h post-inoculation (p≤0.001). Hence, infection with *E*. *histolytica* modified the expression of the *mpo* gene in hamsters (ANOVA, 3(4,20) = 42.3; p<0.001; Fig 5).

### Expression of the *mpo* gene increased in mouse ALAs

Samples of cDNA obtained from mouse ALAs at different post-inoculation times were analyzed by qRT-PCR for quantification of *mpo* gene expression. The results showed that at 3 and 12 h post-inoculation, *mpo* gene expression had a significant increase of ~3.8- fold greater than the basal level (p<0.001). At 6 and 24 h post-inoculation, the value was ~2.30-fold greater than the control group (p<0.001), suggesting that a significantly higher level existed at 3 h than at 6 h post-inoculation (p<0.001) and at 12 h than at 6 or 24 h of evolution (p<0.001). Thus, *E*. *histolytica* infection significantly enhanced the expression of the *mpo* gene in mice (ANOVA, 3(4,20) = 28.747; p<0.001; Fig 5), with the greatest difference observed at 3 and 12 h post-inoculation (p<0.001).

There was a statistically significant difference in the expression of the *mpo* gene between mice and hamsters (p<0.001) (Fig 5). On the one hand, a decrease in *mpo* gene expression was found in hamster ALAs at 3 and 24 h post-inoculation, and an absence of such expression was found at 6 and 12 h. In contrast, an increase in the expression of *mpo* was detected at 3 and 12 h post-inoculation in Balb/c mice ALAs, while a decrease was identified at 6 and 24 h, indicating that the expression of this gene was regulated.

### MPO activity decreased in hamster ALAs and increased in mouse ALA development

**Fig 6:**
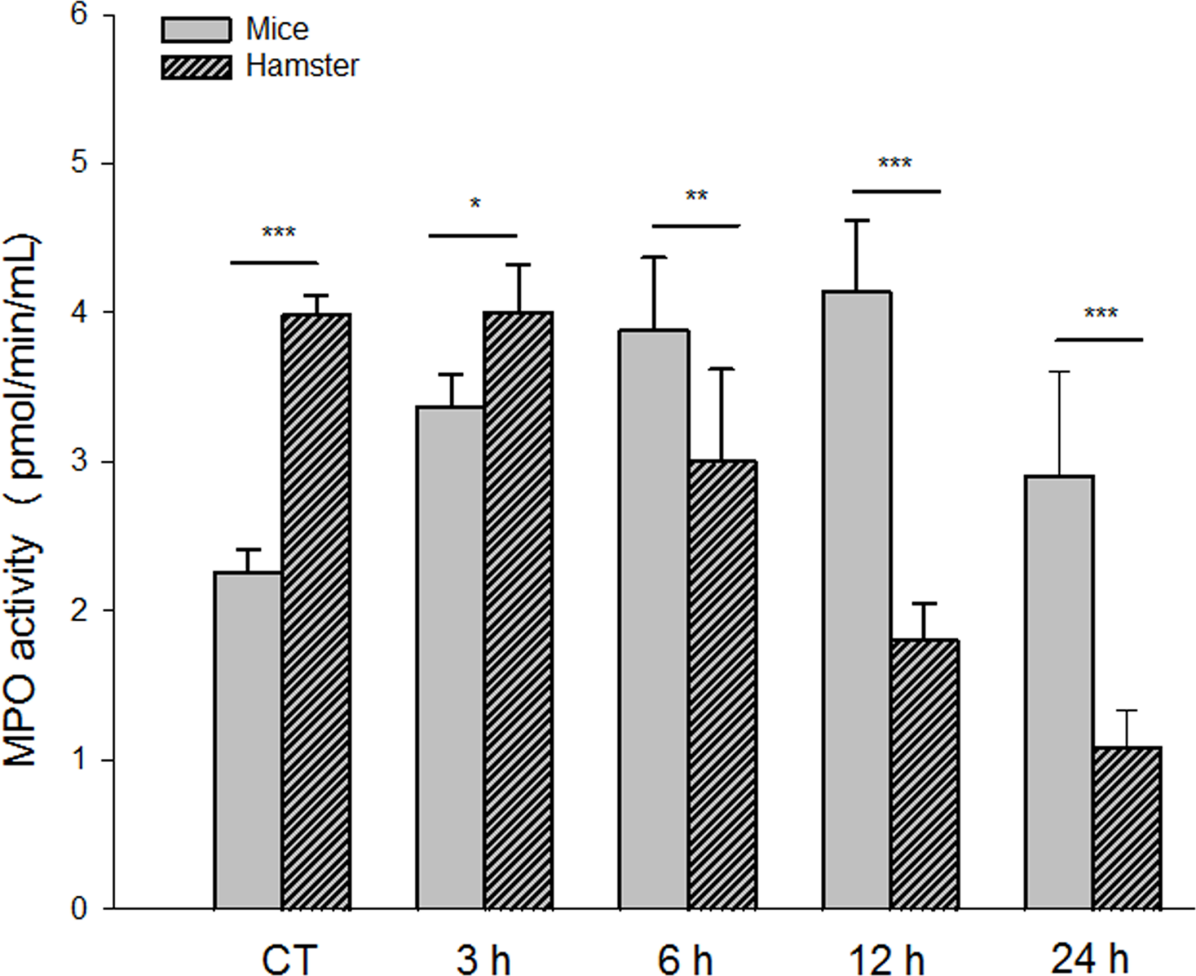
*E*. *histolytica* stimulate the MPO activity in ALA of mice and to decrease in hamsters. Mice tissue samples from ALA showed a significant higher MPO activity from 6–24 h (p<0.001). Hamster liver samples from ALAs showed a significantly reduction of MPO activity from 6 to 24 h (p<0.01). There is a statistically significant difference between the species and post-inoculation time. Data represent the mean ± SD of the three independent experiments, (n = 5). *P*-values were determined by the one-way ANOVA (***p<0.001; ** p<0.01; *p<0.05).

In lysates of hamster ALAs, MPO activity was quantified by a fluorometric method. Hamster MPO activity was modified at different post-inoculation times (ANOVA, 3(4, 20) = 67; p<0.001; Fig 6). At 3 h, the activity was similar compared to the control group [3.98 pmol/min/ml vs 4.06 pmol/min/ml; p = 0.35], followed by a progressive reduction at 6, 12 and 24 h post-inoculation [3.02 pmol/min/ml, 1.8 pmol/min/ml and 1.08 pmol/min/ml, respectively; p<0.001]. Our results showed that in the susceptible model of ALA, MPO activity decreased significantly and in a time-dependent manner, which probably favored the development of hepatic lesions.

In the mouse model, MPO activity was also analyzed by a fluorometric method. There was a gradual and significant increase in a time-dependent manner in MPO activity at 3, 6 and 12 h post-inoculation compared to the control group (ANOVA, 3(4,20) = 14; p<0.001; Fig 6). At 12 h, this value was 4.0 pmol/min/ml versus 2.26 pmol/min/ml in the control. At 24 h, MPO activity diminished to a value of 2.83 pmol/min/ml, almost the same as the level of the control group. MPO activity in mouse ALA showed a gradual increase from 3 to 12 h post-inoculation, suggesting a high concentration of hypochlorous acid. A reduction in MPO activity was found at 24 h.

Comparing MPO activity in the two rodent models, a statistically significant difference existed at all measured times of ALA development at 3, 6, 12 and 24 h (p<0.014, p<0.008, p<0.001 and p<0.001, respectively). MPO activity decreased gradually in hamster ALAs (3–24 h) and increased gradually in mouse ALAs (3–12 h). Overall, the results demonstrate significant differences in MPO activity between mice and hamsters (p<0.001) (Fig 6).

### Effect of the MPO inhibitor on the percentage of ALAs in hamsters and mice

**Fig 7:**
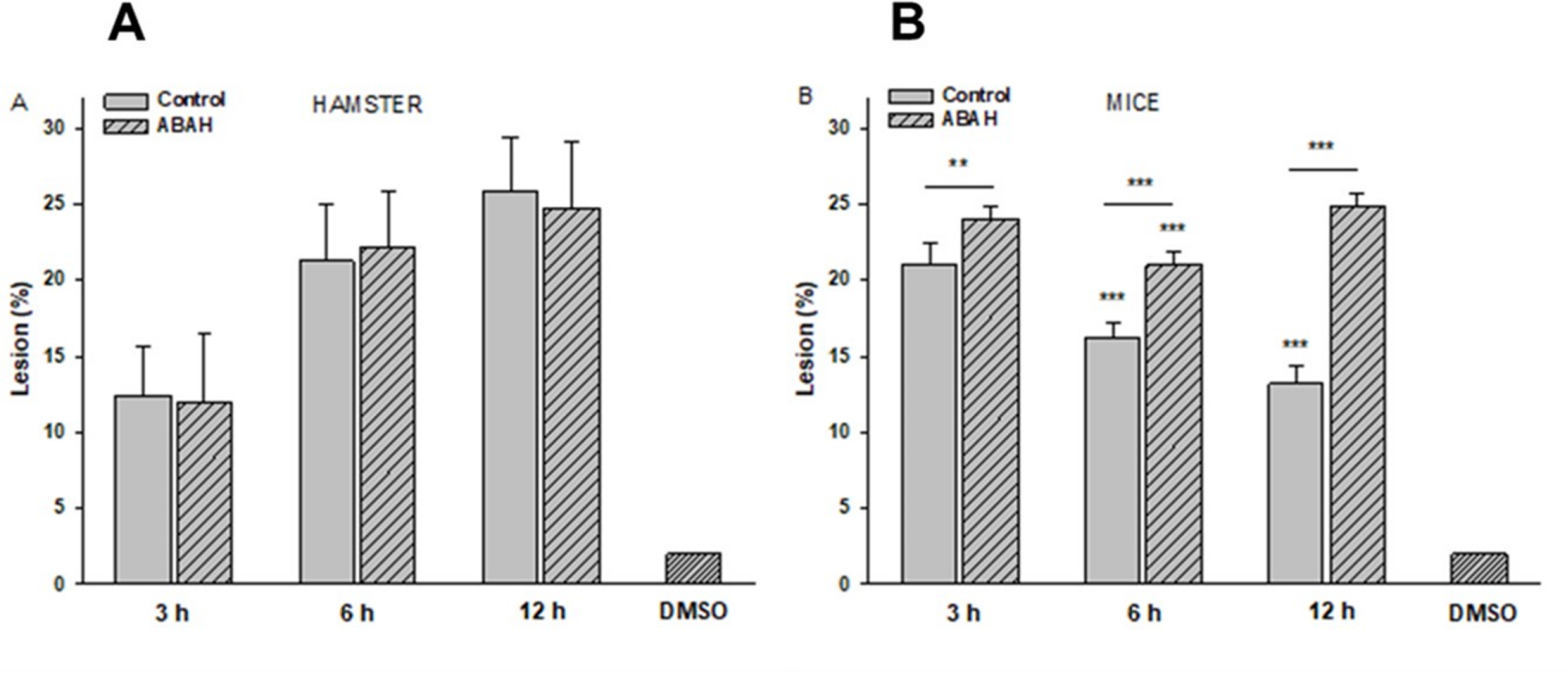
The MPO inhibitor (ABAH) has effect in mouse ALA but not in hamster ALA. Hamsters and mice were treated with 40 mg/Kg of ABAH every 12 h during 5 days, followed by *E*. *histolytica* inoculation and sacrifice at 3, 6, and 12 h post-inoculation. Abscess size is expressed as a percentage of the total liver weight. (A) Hamsters treated with ABAH (diagonal bars) did not present differences in the percentage of lesion with respect to the control group with ABAH (gray bars). (B) Mice treated with ABAH presented a larger size of ALAs at all times tested (diagonal bars). The DMSO control group did not present hepatic damage. Data represent the mean ± SD, (n = 6). *P*-values were determined by the Student’s *t*-test (***p<0.001; ** p<0.01).

In the hamster ALA model, no statistically significant differences were observed in the percentage of lesions between hamsters treated with ABAH and the hamsters not treated. ALA percentages were similar at all post-inoculation times (Fig 7A). For ABAH-treated versus control hamsters (untreatred), the ALA percentage was 12%, versus 13% at 3h, 22% versus 21% at 6 h and 25%, versus 27% at 12 h. In the mouse model clear differences were detected in the percentages of liver damage when treated and untreated animals with the MPO inhibitor (ABAH) were compared. The percentage of ALA was significantly higher in mice treated with the ABAH inhibitor at all post-inoculation times (Fig 7B). For ABAH-treated versus control mice (untreatred), the ALA percentage was 23.4%, versus 21.5% at 3 h, 20.7% versus 15.8% at 6 h and 24.7%, versus 14% at 12 h (p<0.01). The administration of DMSO in the control group did not result in any damage on either animal models.

### The MPO inhibitor did not modify the percentage of damaged *E*. *histolytica* trophozoites in the hamster model

**Fig 8:**
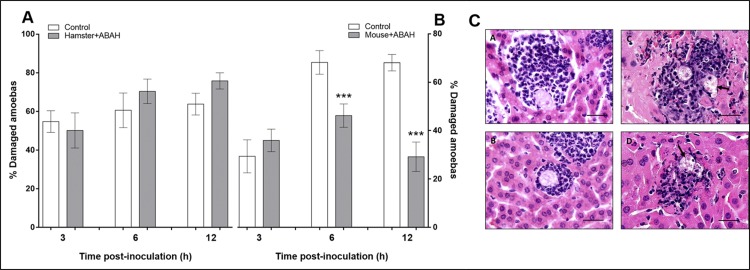
The MPO inhibitor (ABAH) has affect in the percentage of damage amoebae in mouse ALAs but not in amoebae from hamster ALAs. (A) Hamster treated with ABAH not showed significant differences in the percentage of damaged amoebae respect to the control group without ABAH. (B) Mice treated with ABAH showed less percentage of damaged amoebae around 50% at 6 h and 30% at 12 h (that correlates with atypical morphology). Untreated mice presented about 70% of trophozoites damaged at 6 and 12 h. Data represent the mean ± SD (n = 6). *P*-values were determined by Bonferroni *t*-test (***p < 0.001). (C) Undamaged and damaged amoebae in ALA samples stained with H&E. ALA in hamster (A and B) and mice (C and D) at six hours post-inoculation. A and B show undamaged amoebae (with their typical morphology) surrounded by inflammatory cells. C and D show damaged amoebae (arrows) in close contact with the inflammatory cells. Bar = 25 μm.

In hamsters treated and untreated with ABAH, we determined the percentage of damaged amoebae (those showing altered morphology) in amoebic liver lesions. No significant percentage of damaged trophozoites was detected in ABAH-treated hamsters versus untreated hamsters. The percentage of damaged amoeba were similar at all post-inoculation times (Fig 8A).

### MPO inhibitor decreased the percentage of damaged *E*. *histolytica* trophozoites in the mouse model

Significant lower percentage of damaged trophozoites was detected in ABAH-treated mice at 6 and 12 h compared with untreated mice. At 3 h, no significant difference was found between these two groups (Fig 8B) according to the Bonferroni *t*-test (p< 0.001). A representative image of amoebae damaged and undamaged are shown in hamster and mouse ALAs stained with H&E (Fig 8C).

## Discussion

In the present study, we demonstrate for the first time that there was a smaller number of neutrophils positive for MPO in the inflammatory infiltrate of a resistant model of ALA (Balb/c mice), than in a susceptible model (hamsters). However, we observed higher MPO activity and *mpo* gene expression in mice than in hamsters after intraportal inoculation with *E*. *histolytica* trophozoites. Moreover, immunohistochemical analysis showed the presence of MPO in neutrophils in the hepatic lesions of infected mice. There was an increase in cells labeled for MPO in a time-dependent manner. The expression and activity of MPO in the neutrophils of liver parenchyma may have enhanced the cytotoxic capacity of these cells against *E*. *histolytica* trophozoites.

In the present work, our results showed that MPO activity is in accordance with previous studies, in which the increment in the activity of the enzyme is related to higher *mpo* gene expression [49]. During *E*. *histolytica* invasion in the mouse liver parenchyma, it is possible that the MPO enzyme is activated and produces HOCl that can be utilized to eliminate the parasite; therefore, the expression of *mpo* by neutrophils is increased [50]. The present findings suggest a possible protective role for MPO, which could be participating in the elimination of *E*. *histolytica* and consequently preventing the evolution of ALAs in the resistant model (mice).

Additionally, the present study demonstrates for the first time that in a susceptible animal model (hamsters), the number of neutrophils (labeled by AS-D chloroacetate esterase) was elevated at 3 and 6 h of ALA evolution. However, the *mpo* gene expression decreased after the 3 h point of ALA evolution after inoculation with *E*. *histolytica*. This reduction in gene expression was accompanied by diminished MPO activity at 6 h post-inoculation. Hence, the gene expression and enzymatic activity of MPO did not correlate with the number of neutrophils positive for MPO in the hepatic amoebic lesion. This result could be due to the decrease of MPO expression that induces a lower activity of the enzyme, as occurs in multiple sclerosis [51]. Additionally it has been reported that neutrophils may not be sufficiently activated to release MPO or might release an inactive MPO, as reported in other pathologies. In all these conditions, the cytotoxic potential of neutrophils would not be reached [31, 35],[33, 52, 53].

The MPO protein was present in neutrophils located in amoebic hepatic lesions, which could be related to the findings of a previous study reporting that MPO is produced in the early stages of the development of myeloid leukemia and is stored in the cytoplasmic vesicles of neutrophils and monocytes [54]. In neutrophils, MPO constitutes 2 to 5% of total proteins [55]. The presence of MPO in hamster ALAs is perhaps due to the incapacity of the neutrophils to release it, which would explain the diminished activity of the enzyme that would in turn prompt a significant reduction in its expression [54]. Further research is necessary on this topic.

The immunohistochemical assay indicated the presence of MPO in the inflammatory reaction and in the necrotic areas of ALAs in both animal models. Nevertheless, the semi-quantitative analysis of positive cells for MPO revealed differences between these two rodents. In the mouse model, cells positive for MPO significantly increased at 6 and 12 h.

On the other hand, in the hamster model, there was a significant decrease in cells positive for MPO at 12 and 24 h post-inoculation with *E*. *histolytica* trophozoites.

It is important to note that differences exist between these two models in the number of neutrophils present in ALAs, which is consistent with previous reports [7, 8, 56]. Additionally, the gene expression and enzymatic activity of MPO was different. Nevertheless, the evolution of ALAs in hamsters is probably related to the decrease in MPO-positive neutrophils and to the relatively low enzymatic activity. This phenomenon could be related to the resolution of ALAs in the resistant animals or to the development of ALAs in hamsters.

The existence of polymorphisms in the promoter region of the *mpo* gene may be one possible explanation for the differences in MPO found herein between the two animal models. Various studies have demonstrated that polymorphisms in this region affect gene function and increase susceptibility to various inflammatory diseases, such as chronic granulomatous disease [57–60]. In the susceptibility model of ALA, there might be polymorphisms modifying the expression of the promoter elements that regulate the expression of the *mpo* gene in neutrophils [61], as well as factors of virulence in amoebae, such as lectin Gal/GalNac, amoebapore and cysteine proteases [62], GalNac [63], proteasas [64–66], amebopore [67, 68].

The differences between the susceptible and resistant animal models in regard to MPO activity, protein level and gene expression could also be due to the presence of endogenous inhibitors of this enzyme in hamsters. *In vivo* studies have suggested that MPO may be blocked by endogenous inhibitors such as ceruloplasmin, which would explain why MPO activity does not always correspond to gene expression or protein levels [52, 69]. It is also necessary to keep in mind that the hamster ALA model presents an exacerbated inflammation [70–74], and an overproduction of reactive oxygen intermediates like HOCl produced by MPO and to avoid the host tissue damage by these molecules, the immune system modulates the production of radicals through the antioxidant enzymes that could inhibit the expression of several proinflammatory genes including the MPO enzyme, among others [75, 76]. However, these mechanisms need to be explored in hamsters. On the one hand, some studies have demonstrated that the partial inhibition of MPO activity is beneficial because this enzyme is implicated in regulating the positive feedback of inflammation [18–21]. Moreover, MPO has inhibitory effects on the function of lymphocytes and can suppress inflammation through the inactivation of soluble chemotactic factors such as C5a. Therefore, MPO modulation of aspects of the inflammatory response may depend on the environment [77, 78]. For example, after zymosan-induced inflammation in the lungs of MPO-deficient mice [79], MPO administration attenuated lung damage caused by inflammation. Another report suggests that MPO has a dual role, first provoking an inflammatory response to the oxidative degradation of foreign material, and then eventually attenuating and regulating inflammation [80].

The low enzymatic activity for the longer times (6 to 24 h) and the low *mpo* gene expression in neutrophils in the hamster model would allow the amoebae to remain viable and allow the ALA to evolve. As aforementioned, it is known that neutrophils deficient in MPO have a diminished capacity to eliminate pathogens such as *Naegleria fowleri* [38]. Moreover, a high susceptibility to infection with *Klebsiella pneumoniae* was described for animals deficient in MPO, and these animals eventually died as a result of the infection [23]. Various studies have concluded that MPO activity eliminates not only intracellular pathogens, but also extracellular organisms such as *Strongyloides stercoralis* larva [38, 40, 41, 81].

On the other hand, it is important to mention that the interaction between the host immune response and the amoebae molecules have an important role in determining resistance or susceptibility to the evolution of ALA. In the hamster model, several authors have reported that the strong inflammatory reaction participate in the liver damage. In the mouse, an adequate immune response, resolve the liver lesion and the inflammation can eliminate the amoebae [7, 8, 11].

When the ABAH, an irreversible MPO inhibitor was inoculated into hamsters no significant differences between hamsters treated with ABAH versus the control group were observed, but different inhibitor concentrations should be tested in this model. Regarding the current results in the resistant model, MPO could possibly have participated in the elimination of the amoeba and at the same time regulated inflammation independently of its enzymatic activity [19, 82], therefore avoiding severe damage to the liver. Consequently, we investigated the role of MPO in the resistant ALA model by using ABAH. When comparing mice treated and untreated with ABAH, the former showed larger ALAs post-inoculation with *E*. *histolytica*. Hence, it is probable that MPO inhibition favored the development of ALA, which suggests that the MPO enzyme plays a protective role by participating in the destruction of *E*. *histolytica*, an idea corroborated by the significant reduction in the percentage of damaged amoebae in the ALA of mice treated with ABAH. A similar effect of this MPO inhibitor has been previously documented, as in infections with *Besnoitia besnoiti* [83].

Although we observed inflammation in the liver for both the susceptible and resistant animal models, our hypothesis is that MPO activity could have an amebicidal function in the mice. It is also possible that MPO binds monocytes and stimulates the production of reactive oxygen or nitrogen species [84], which would participate in amoebic damage; such a possibility should be explored in future studies.

We can conclude that the presence of MPO in Balb/c mice may favor the positive resolution of amoebic liver damage, and that the reduction or absence of this enzyme in hamsters allows for the development of ALA. Moreover, amoebic factors or molecules may inhibit or block the expression and activity of MPO in the susceptible model, thus favoring ALA development in hamsters. Also it is important to evaluate the MPO activity in other cell types as monocytes. Further research is required to elucidate the mechanisms that are possibly involved in a protective role of MPO against the proliferation of *E*. *histolytica* trophozoites in the resistant model. For example, it is possible that MPO has a dual role, modulating inflammation while exhibiting an amoebicidal effect.

## Supporting information

S1 Fig ALA samples stained with hematoxylin & eosin. ALA in hamster (A and B) and mice (C and D) at six hours post-inoculation. In both animals are observed an inflammatory reaction with several neutrophils. B y D showed the classical morphology of neutrophils. A and C Barr = 10μm; B and D Barr = 25μm.
(TIF)

S2 Fig A higher magnification of ALAs from hamsters and mice with AS-D chloroacetate esterase. Neutrophils show their characteristic morphology and label to AS-D esterase. A and C ALA in hamster. B and D ALA from mice. Bar = 10 μm.
(TIF)

S3 Fig Immunofluorescence of MPO and anti-amoeba in hamster ALAs. (A) ALA at 6 h post-inoculation, two amoebae (arrows) is seen in two inflammatory foci positive to anti-amoeba antibody (red). MPO FITC (arrowheads) was observed in the inflammatory infiltrate revealing the presence of MPO. Hepatocytes were no stained to MPO (H). (B) Phase contrast microscopy of ALA from two inflammatory foci with the presence of trophozoites.
(TIF)
