# Supplementary figures and images for: Different behavior of myeloperoxidase in two rodent amoebic liver abscess models
Source: PLoS One. 2017 Aug 10;12(8):e0182480. doi: 10.1371/journal.pone.0182480 (PMC5552100; doi:10.1371/journal.pone.0182480)

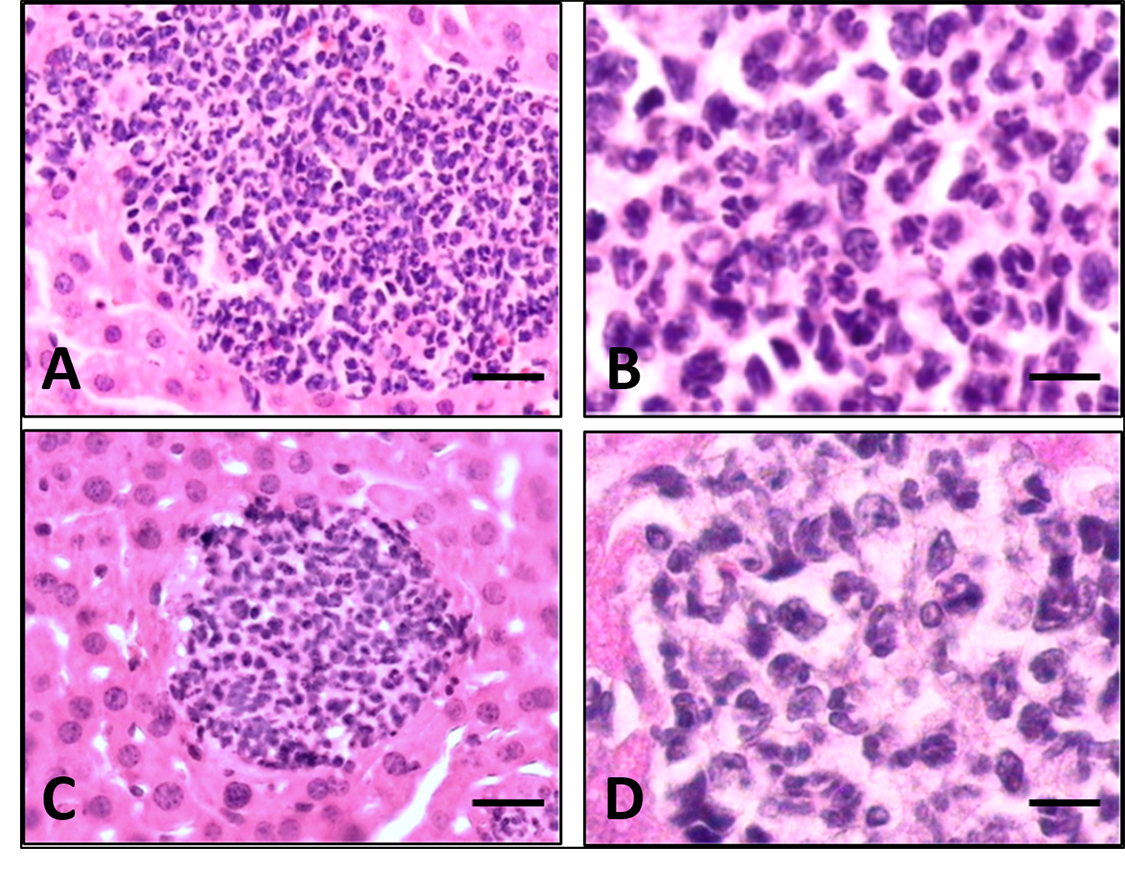

Supplement: S1 Fig — ALA in hamster (A and B) and mice (C and D) at six hours post-inoculation. In both animals are observed an inflammatory reaction with several neutrophils. B y D showed the classical morphology of neutrophils. A and C Barr = 10μm; B and D Barr = 25μm. (TIF) [file pone.0182480.s001.tif]

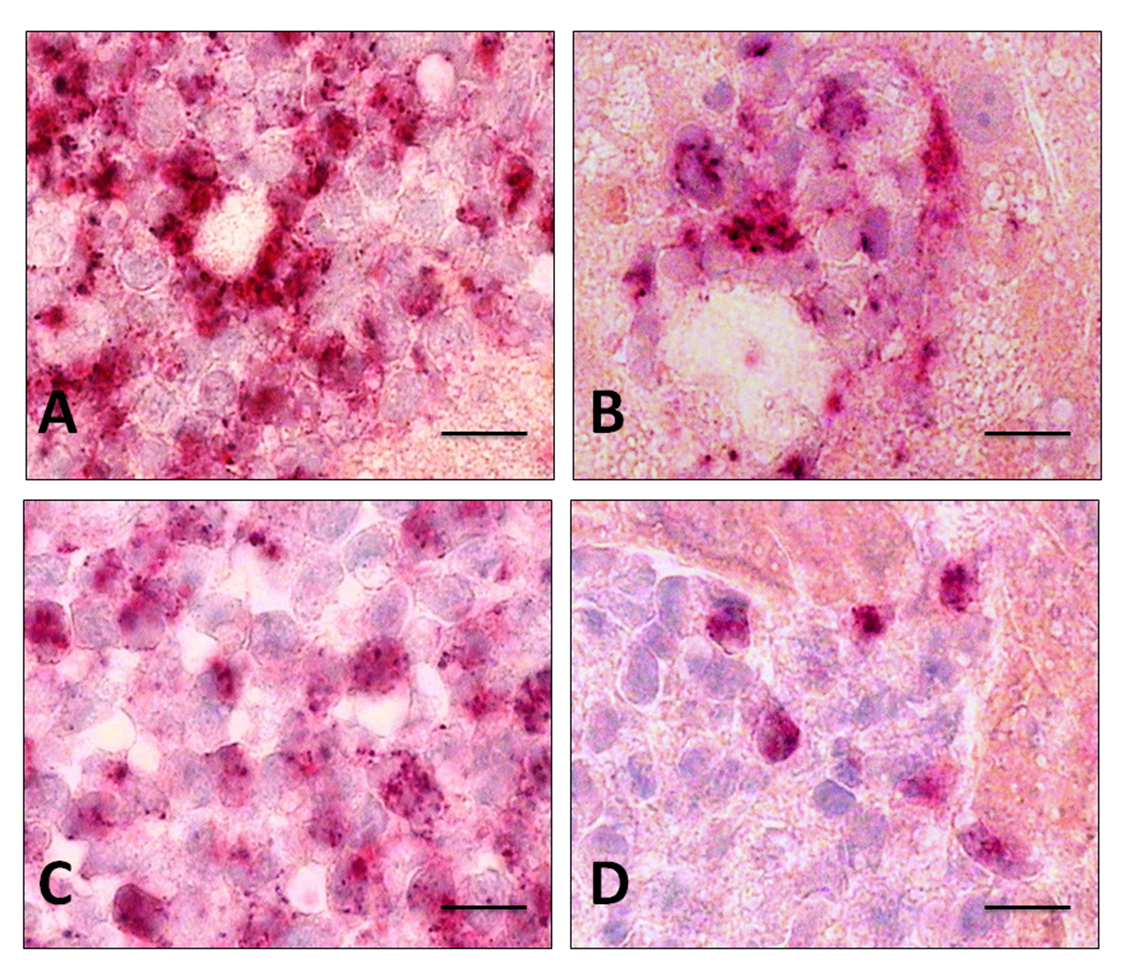

Supplement: S2 Fig — Neutrophils show their characteristic morphology and label to AS-D esterase. A and C ALA in hamster. B and D ALA from mice. Bar = 10 μm. (TIF) [file pone.0182480.s002.tif]

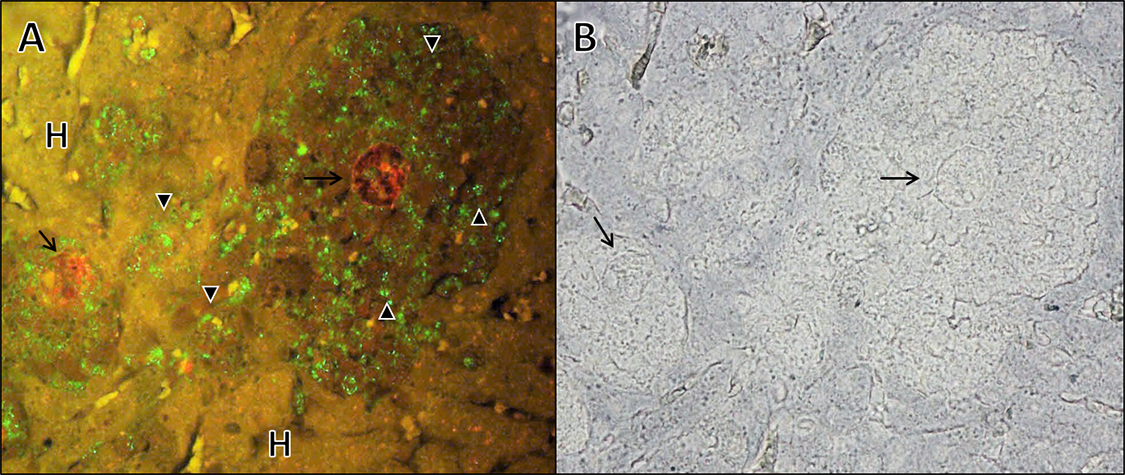

Supplement: S3 Fig — (A) ALA at 6 h post-inoculation, two amoebae (arrows) is seen in two inflammatory foci positive to anti-amoeba antibody (red). MPO FITC (arrowheads) was observed in the inflammatory infiltrate revealing the presence of MPO. Hepatocytes were no stained to MPO (H). (B) Phase contrast microscopy of ALA from two inflammatory foci with the presence of trophozoites. (TIF) [file pone.0182480.s003.tif]
